# Drip loss assessment by EZ and bag methods and their relationship with pH value and color in mutton

**DOI:** 10.5194/aab-63-277-2020

**Published:** 2020-08-26

**Authors:** Ana Kaić, Ante Kasap, Ivan Širić, Boro Mioč

**Affiliations:** Department of Animal Science and Technology, University of Zagreb, Faculty of Agriculture, 10000 Zagreb, Croatia

## Abstract

Drip loss, pH value, and color are among the important traits that determine meat quality. Contrary to pH and color, the method associated with drip loss is not yet standardized, and literature data are difficult to compare. Besides, to our knowledge, there is no research comparing drip loss methods and their relation with pH and color in mutton. This study aimed to assess drip loss measurements in mutton taken by different methods (EZ and bag – BM) and their relationship with pH values and color. Mutton samples (*Musculus longissimus thoracis et lumborum*) originating from 20 ewes of Istrian sheep were used to examine the effect of the method on drip loss after 24 h (EZ${}_{24}$ vs. BM${}_{24}$) and 48 h (EZ${}_{48}$ vs. BM${}_{48}$). Furthermore, correlations between drip loss, pH value, and color were analyzed. The statistical analysis was conducted in R programming environment by using different packages. Within the EZ method there was no significant difference (p>0.05) between ventral and dorsal sample cores used for the assessment of EZ drip loss. Drip loss measured with the same method at two different points of time (24 and 48 h) differed significantly (p<0.001). There was also a significant difference in drip loss determined by different methods (EZ vs. BM) at the same point of time. There were significant (p<0.05) correlations between pH${}_{45\;\min}$ and all color parameters ($L^{*}4$, $a^{*}$, $b^{*}$). The $L^{*}$, $a^{*}$, and $b^{*}$ parameters were highly correlated (p<0.001). The strongest correlation occurred between $a^{*}$ and $b^{*}$ parameter (r=0.93). Correlations between drip loss by EZ method and other meat quality attributes were low and not significant. The $b^{*}$ parameter correlated with BM${}_{24}$ (r=0.46) and BM${}_{48}$ (r=0.58), while $a^{*}$ correlated only with BM${}_{48}$ (r=0.50). The correlations between the EZ${}_{24}$ and BM${}_{24}$ as well as between the EZ${}_{48}$ and BM${}_{48}$ were both non-significant (p>0.05). Drip loss cannot be predicted with sufficient accuracy by using pH and color. EZ and BM method in mutton do not provide equivalent results for measuring drip loss. Comparisons of the results obtained with different methods should be avoided or at least performed with great precaution.

## 1 Introduction

Considering numerous traits that determine meat quality, drip loss, pH value, and color are among the important ones associated with consumer acceptance and processing technology. It is known that rapid pH decline during rigor development may lead to protein denaturation related to color, tenderness, and water-holding capacity (Kim et al., 2014). Color is considered the main factor in consumer acceptance and purchasing of different types of meat (Arshad et al., 2018). High drip loss values result in numerous losses (appearance, nutritional value, texture parameters, and attractiveness), thereby affecting the quality of fresh meat and its different products (Otto et al., 2004). The two most widely utilized methods for measuring drip loss are the bag method and the EZ method (Mason et al., 2016). They are gravimetric methods in which the meat is suspended in a container for drip usually 24 or 48 h, and the only force on the meat is gravity. The bag method is performed with cubed samples of 40–100 g, whereas the EZ method uses cylindrical samples of 5–10 g (Rasmussen and Andersson, 1996; Honikel, 1998). However, this method of drip loss determination has not yet been standardized, and literature data are difficult to compare. Besides, to our knowledge, there is no research comparing drip loss methods in mutton. Therefore, this study aimed to assess drip loss measurements in mutton taken by EZ and bag methods and their relationship with pH values and color.

## 2 Material and methods

### 2.1 Animals, slaughtering, and sampling

The study was conducted on mutton samples originating from 20 culled ewes from Istrian sheep. The ewes were reared in a semi-intensive dairy production system and were culled from the flocks when their milk production fell below the acceptable level. The average age of the animals was 87 months, with a range from 35 to 116 months. The animals were slaughtered and processed under the normal conditions following the guidelines set out in Council Regulation (EC) No. 1099/2009 (European Communities, 2009) on the protection of animals at the time of killing. After the slaughtering procedure and evisceration process, the carcasses were chilled at 4 ${}^{\circ }$C for 24 h in a cold chamber. Muscle samples for the analysis were taken from the loin (*M. longissimus thoracis et lumborum* – LL) of each carcass at 24 h post-mortem. The LL was removed from the cranial edge to the 12th or 13th rib. After that, the samples were transported to the laboratory for further sectioning and analysis. The aforementioned procedures were conducted according to the guidelines of EU Directive 2010/63/EU (2010) on the protection of animals used for experimental and other scientific purposes.

### 2.2 Analytical methods

The pH values of the LL muscle were measured at 45 min (pH${}_{45\;\min}$) post-mortem between the 12th and 13th thoracic vertebrae, using a penetrating electrode (Schott BlueLine 21pH attached to a portable pH meter IQ 150, Scientific Instruments, USA). Meat color parameters ($L^{*}$ – lightness, $a^{*}$ – redness, and $b^{*}$ – yellowness) were successively measured on the cross section of the LL muscle after a 1 h blooming period using a chroma meter (Konica Minolta Chroma Meter CR 400, Osaka, Japan). Drip loss was measured according to the EZ method (Rasmussen and Andersson, 1996) and bag method – BM (Honikel, 1998). For determination of drip loss according to BM, the 60 g of sample was removed from the cranial edge of the LL muscle. The samples for the BM were weighed and then suspended separately in an inflated bag. The EZ drip loss method was carried out on the sample of 20 mm thickness, followed after removal of the samples for the BM. A two cylindrical muscle core samples, at dorsal and ventral position, were removed using a circular knife (Ø 25 mm × 20 mm height). These samples were weighed and after that placed within specialized EZ drip loss containers. Drip loss assessment by BM and EZ methods was performed after a storage period of 24 and 48 h at 4 ${}^{\circ }$C, as the change in sample weight was expressed as a percentage. Before each final weighing, there was no need for dabbing of the muscle surface samples.

### 2.3 Statistical analysis

The statistical analysis was conducted in R programming environment by using different packages. Descriptive statistics were obtained with package “pastecs” (Grosjean and Ibanez, 2018), boxplots with “graphics” (R Core Team, 2018), and correlations with “Hmisc” (Harrell, 2019). The effect of different methods (EZ${}_{24}$ vs. BM${}_{24}$ and EZ${}_{48}$ vs. BM${}_{48}$) and anatomical position of the muscle on drip loss (EZ${}_{24\_V}$ vs. EZ${}_{24\_D}$, EZ${}_{48\_V}$ vs. EZ${}_{48\_D}$) were examined with paired t tests. In the analysis of the effect of methods on drip loss, the values for EZ${}_{24}$ and EZ${}_{48}$ were obtained by averaging EZ${}_{24\_V}$ and EZ${}_{24\_D}$ as well as EZ${}_{48\_V}$ and EZ${}_{48\_D}$, respectively. Shapiro–Wilk normality tests of pair-wise differences and paired t tests for the above-discussed scenarios were conducted with package “stats” (R Core Team, 2018).

**Table 1:** Means ($\bar{x}$) with standard error (SE), minimum (Min), maximum (Max), and coefficient of variation (CV) for meat quality attributes of mutton (n=20).

Attribute	$\bar{x}$	SE	Min	Max	CV, %
pH${}_{45\;\min}$	6.11	0.06	5.55	6.62	4.41
$L^{*}$	31.39	0.41	28.94	35.06	5.87
$a^{*}$	17.69	0.43	14.59	22.19	11.10
$b^{*}$	2.27	0.22	0.82	4.36	43.95
EZ${}_{24\_V}$ (%)	0.65	0.09	0.02	1.69	66.60
EZ${}_{24\_D}$ (%)	0.66	0.10	0.01	1.37	68.64
EZ${}_{24}$ (%)	0.65	0.09	0.02	1.53	65.84
EZ${}_{48\_V}$ (%)	0.91	0.09	0.17	1.70	44.83
EZ${}_{48\_D}$ (%)	0.94	0.11	0.24	1.83	52.14
EZ${}_{48}$ (%)	0.93	0.10	0.21	1.73	47.68
BM${}_{24}$ (%)	1.46	0.07	0.99	2.20	23.06
BM${}_{48}$ (%)	2.26	0.13	1.40	3.22	27.14

pH${}_{45\;\min}$ – pH values of the LL muscle measured at 45 min post-mortem, $L^{*}$ – lightness, $a^{*}$ – redness, $b^{*}$ – yellowness, EZ${}_{24\_V}$ – EZ drip loss by weighing samples after 24 h storage in the ventral position, EZ${}_{24\_D}$ – EZ drip loss by weighing samples after 24 h storage in the dorsal position, EZ${}_{24}$ – EZ drip loss obtained by averaging EZ${}_{24\_V}$ and EZ${}_{24\_D}$, EZ${}_{48\_V}$ – EZ drip loss by weighing samples after 48 h storage in the ventral position, EZ${}_{48\_D}$ – EZ drip loss by weighing samples after 48 h storage in the dorsal position, EZ${}_{48}$ – EZ drip loss obtained by averaging EZ${}_{48\_V}$ and EZ${}_{48\_D}$, BM${}_{24}$ – drip loss by the bag method after 24 h storage, BM${}_{48}$ – drip loss by the bag method after 48 h storage.

## 3 Results and discussion

### 3.1 Relationship between drip loss values measured by EZ method

**Figure 1:**
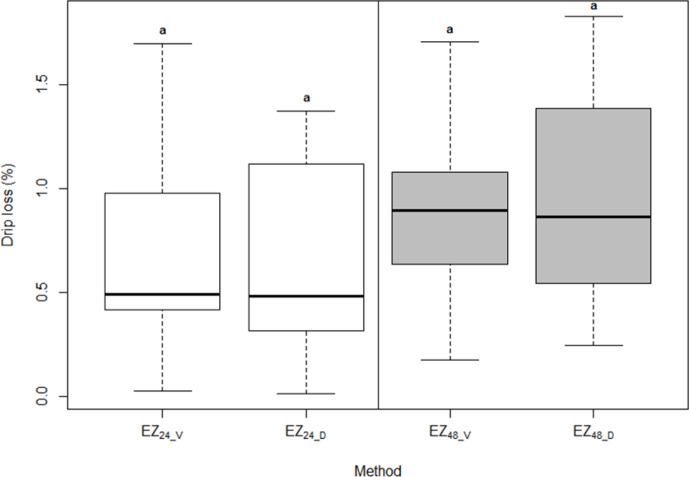
Distributions of the drip loss obtained on ventral and dorsal cores with EZ method after 24 and 48 h. For abbreviations see Table 1. Letters represent the results of paired t test with significance level p<0.05.

The mean value for drip loss measured at the ventral side after 24 h was 0.65 %, and the dorsal side was 0.66 %. The mean value for drip loss measured at ventral side after 48 h was 0.91 %, and the dorsal side was 0.94 % (Table 1). Distributions of the measurements obtained on ventral and dorsal cores suggested that there were no significant differences between the sampling site, which was confirmed with the paired t test (p>0.05; Fig. 1). This result was probably due to a high degree of homogeneity of the LL muscle that was detected visually during the sampling procedure. Uniform visual appearance during the sampling procedure, along with the obtained results, implies that taking two sample cores is redundant in the drip loss analysis of the mutton LL muscle. Contrary to our results, in porcine meat Christensen (2003) and Otto et al. (2004) reported variations in the EZ method within the results due to the sampling position, indicating that this factor must be considered. They found significantly higher drip loss in the ventral part of the *longissimus dorsi* muscle than in the dorsal part.

### 3.2 Relationship between drip loss values measured by EZ method and BM

**Figure 2:**
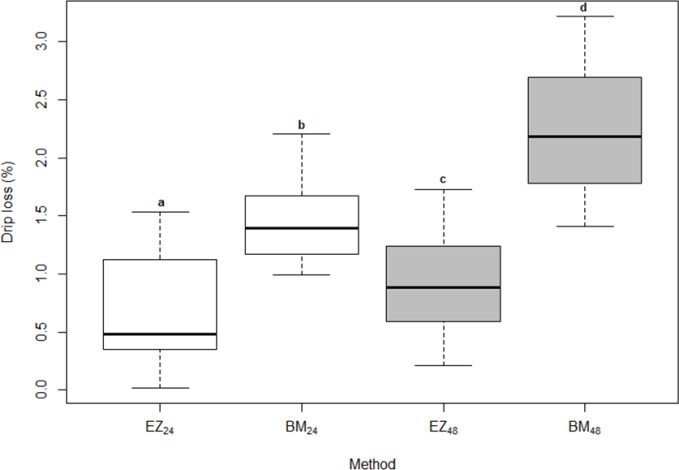
Distributions of drip loss obtained by BM and EZ methods after 24 and 48 h. For abbreviations see Table 1. Letters represent the results of paired t test. Different letters in the same row significantly differ (p<0.001).

Figure 2 shows distributions of drip loss values obtained by the EZ method and BM after 24 and 48 h. As suggested by Rasmussen and Andersson (1996), the mean value of dorsal and ventral muscle samples was used for the assessment of the EZ method. The mean values for drip loss measured by the BM after 24 h (1.46 %) and 48 h (2.26 %) were higher than for the EZ method (0.65 % for 24 h and 0.93 % for 48 h; Table 1). Drip loss measured with the same method at two different points of time (24 and 48 h) differed significantly (p<0.001; Fig. 2). There was also a significant difference (p<0.001; Fig. 2) in drip loss determined by different methods (EZ vs. BM) at the same point of time. The results are in agreement with Christensen (2003) and Filho et al. (2017), who also noticed higher drip loss values using the BM compared to the EZ method. On the contrary, Honikel and Hamm (1994), Christensen (2003), and Otto et al. (2004) found higher drip loss values using the EZ method. These differences were explained by the greater surface area to weight ratio of the samples used in the EZ method. However, Filho et al. (2017) did not observe higher drip loss values for the EZ samples despite their greater surface area to weight ratio compared to BM (4.6 vs. 1.8). They emphasized that the surface area in which water primarily escapes is more important. Furthermore, the direction of the muscle fibers in the samples used for the EZ method is vertical, whereas for the BM it is horizontal and could be the reason for higher drip loss values found in BM. Concerning this, Holman et al. (2020), reported no differences in EZ drip loss of the *semimembranosus* muscle using horizontal and vertical sample fiber orientations. They explained that smaller drip loss sample size had lesser physical resistance for immobilization of this water fraction as it transverses the meat structural matrix and may have overcome any fiber orientation to drip loss variation of the samples.

The results showed that the samples stored for 48 h had significantly higher drip loss than those stored for 24 h. Since it is known that the exudation in the muscle is a complex and slow process, this was somehow expected. Otto et al. (2004), Correa et al. (2007), Filho et al. (2017), and Holman et al. (2020) also confirmed this tendency for drip loss to increase with storage period. The difference in mean values for the EZ method in the present study between 24 and 48 h of storage was 0.28 %, whereas for the same period of time for BM it was 0.80 %.

**Table 2:** Correlation coefficients among meat quality attributes of mutton (n=20)${}^{a}$.

	pH${}_{45\;\min}$	$L^{*}$	$a^{*}$	$b^{*}$	EZ${}_{24}$ (%)	EZ${}_{48}$ (%)	BM${}_{24}$ (%)
$L^{*}$	-0.50${}^{*}$						
$a^{*}$	-0.46${}^{*}$	0.85${}^{***}$					
$b^{*}$	-0.47${}^{*}$	0.88${}^{***}$	0.93${}^{***}$				
EZ${}_{24}$ (%)	-0.14	0.02	0.05	-0.04			
EZ${}_{48}$ (%)	-0.23	0.16	0.08	0.08	0.93${}^{***}$		
BM${}_{24}$ (%)	-0.32	0.30	0.43	0.46${}^{*}$	-0.41	-0.27	
BM${}_{48}$ (%)	-0.29	0.30	0.50${}^{*}$	0.58${}^{**}$	-0.35	-0.25	0.70${}^{***}$

${}^{a}$ For abbreviations see Table 1. ${}^{*}$ p<0.05. ${}^{**}$ p<0.01. ${}^{***}$ p<0.001.

### 3.3 Correlations among meat quality attributes

The correlation coefficients between drip loss, pH, and color values are presented in Table 2. The study revealed significant (p<0.05) intermediate negative correlations (from -0.46 to -0.50) between pH${}_{45\;\min}$ and all color parameters ($L^{*}$, $a^{*}$, $b^{*}$) suggesting that the increase of pH is accompanied by a decrease of all color parameters.

The $L^{*}$, $a^{*}$, and $b^{*}$ color parameters were mutually highly correlated (r>0.85, p<0.001). The strongest relationship occurred between $a^{*}$ and $b^{*}$ parameters (r=0.93). The results are in line with the report of Page et al. (2001), who also found the strongest correlation between $a^{*}$ and $b^{*}$ values (r=0.95). Within this finding, they indicated that $a^{*}$ is probably more useful than $b^{*}$ when measuring color stability because $a^{*}$ is a value from red to green, and surface metmyoglobin formation changes the color from red to greenish-brown.

Correlations between drip loss by EZ method and other meat quality attributes were low and non-significant. Contrary to that, it was found that $b^{*}$ value correlates with BM${}_{24}$ (r=0.46) and BM${}_{48}$ (r=0.58), while $a^{*}$ value correlates only with BM${}_{48}$ (r=0.50). The correlation between $L^{*}$ and drip loss (EZ${}_{24}$, EZ${}_{48,}$ BM${}_{24}$, BM${}_{48}$) was positive (but low and non-significant), which is in general agreement with theoretical expectations on this issue (Guo and Dalrymple, 2017). High correlations were determined between drip loss EZ${}_{24}$ and EZ${}_{48}$ (r=0.93) and somewhat lower between BM${}_{24}$ and BM${}_{48}$ (r=0.70), which was reasonable (due to the repeated measurements on the same samples). The correlations between drip loss obtained by using the EZ${}_{24}$ and BM${}_{24}$ or BM${}_{48}$ was negative and intermediate but non-significant (r=-0.41 vs. r=-35). A similar relationship was also found between drip loss obtained by using the EZ${}_{48}$ and BM${}_{24}$ or BM${}_{48}$ (r=-0.27 vs. r=-0.25). The aforementioned correlations suggest that the EZ method and BM in mutton do not provide equivalent results for measuring drip loss. However, Otto et al. (2004) found a high relationship between EZ${}_{48}$ drip loss with BM${}_{24}$ or BM${}_{48}$ (r=0.86) in porcine meat. In addition to this, there are several studies on porcine meat (Otto et al., 2006; Correa et al., 2007) and alpaca meat (Logan et al., 2019) suggesting that both methods are reliable in drip loss assessment.

## 4 Conclusions

Different sampling sites of the LL muscle in mutton provided very similar EZ drip loss values, implying that sampling on both sides of the muscle is redundant and does not contribute too much to the accuracy of the analysis. The color and pH value of meat are insufficiently informative for accurate prediction of drip loss in the mutton. A discrepancy in the drip loss obtained with the different methods indicates that results of the drip loss in mutton are heavily dependent on the method. Comparisons of the results obtained with different methods should be avoided or at least performed with great precaution.

## Data Availability

The data from this study can be accessed from the corresponding author upon reasonable request.

## References

[bib1.bib1] Arshad MS, Sohaib M, Ahmad RS, Nadeem MT, Imran A, Arshad MU, Kwon J, Amjad Z (2018). Ruminant meat flavor influenced by different factors with special reference to fatty acids. Lipids Health Dis.

[bib1.bib2] Christensen LB (2003). Drip loss sampling in porcine *m. longissimus dorsi*. Meat Sci.

[bib1.bib3] Correa JA, Méthot S, Faucitano L (2007). A modified meat juice container (EZ-DripLoss) procedure for a more reliable assessment of drip loss and related quality changes in pork meat. J Muscle Foods.

[bib1.bib4] (2010). Directive 2010/63/EU of the European Parliament and of the Council of 22
September 2010 on the protection of animals used for scientific purposes. Official Journal of the European Union.

[bib1.bib5] European Communities (2009). Council Regulation (EC) No. 1099/2009 of 24 September 2009 on the protection of
animals at the time of killing. Official Journal of the European Communities.

[bib1.bib6] Filho RDAT, Cazedey HP, Fontes PR, Ramos ADLS, Ramos EM (2017). Drip loss assessment by different analytical methods and their relationships with pork quality classification. J Food Qual.

[bib1.bib7] Grosjean P, Ibanez F (2018). pastecs: Package for Analysis of Space-Time Ecological Series.

[bib1.bib8] Guo B, Dalrymple BP, Purslow, PP (2017). Transcriptomic of meat quality. New Aspects of Meat Quality From Genes to Ethics.

[bib1.bib9] Harrell Jr. FE (2019). Hmisc: Harrell Miscellaneous.

[bib1.bib10] Holman BWB, Alvarenga TIRC, Hopkins DL (2020). The effect of fibre orientation, measurement interval and muscle on lamb meat drip loss values. Meat Sci.

[bib1.bib11] Honikel KO (1998). Reference methods for the assessment of physical characteristics of meat. Meat Sci.

[bib1.bib12] Honikel KO, Hamm R, Pearson AM, Dutson, TR (1994). Measurement of water-holding capacity and juiciness. Quality Attributes and Their Measurement in Meat, Poultry and Fish Products.

[bib1.bib13] Kim YHB, Warner RD, Rosenvold K (2014). Influence of high pre-rigor temperature and fast pH fall on muscle proteins and meat quality: a review. Anim Prod Sci.

[bib1.bib14] Logan BG, Bush RD, Biffin TE, Hopkins DL, Smith MA (2019). Measurement of drip loss in alpaca (Vicugna pacos) meat using different techniques and sample weights. Meat Sci.

[bib1.bib15] Mason A, Abdullah B, Muradov M, Korostynska O, Al-Shamma'a A, Bjarnadottir SG, Lunde K, Alvseike O (2016). Theoretical basis and application for measuring pork loin drip loss using microwave spectroscopy. Sensors.

[bib1.bib16] Otto G, Roehe R, Looft H, Thoelking L, Kalm E (2004). Comparison of different methods for determination of drip loss and their relationships
to meat quality and carcass characteristics in pigs. Meat Sci.

[bib1.bib17] Otto G, Roehe R, Looft H, Thoelking L, Henning M, Plastow FS, Kalm E (2006). Drip loss of case-ready meat and of premium cuts and their associations with earlier
measured sample drip loss, meat quality and carcass traits of pigs. Meat Sci.

[bib1.bib18] Page JK, Wulf DM, Schwotzer TR (2001). A survey of beef muscle color and pH. J Anim Sci.

[bib1.bib19] R Core Team (2018). R: A language and environment for statistical computing.

[bib1.bib20] Rasmussen AJ, Andersson M (1996). New method for determination of drip loss in pork muscles.

